# Tumor microenvironment in Hodgkin lymphoma: novel prognostic factors for assessing disease evolution

**DOI:** 10.25122/jml-2023-0239

**Published:** 2023-08

**Authors:** Andrei Turbatu, Camelia Dobrea, Marilena Stoian, Cristian Tudor Barta, Georgian Halcu, Adelina Birceanu, Ana-Maria Bordea, Cecilia Gabriela Ghimici, Mădălina Marilena Oprea, Livia Doria Neacșu, Anca-Roxana Lupu, Andrei Coliță

**Affiliations:** 1 Department of Hematology, Carol Davila University of Medicine and Pharmacy, Bucharest, Romania; 2 Clinic of Hematology, Colțea Clinical Hospital, Bucharest, Romania; 3 OncoTeam Diagnostic Laboratory, Royal Hospital Clinic, Bucharest, Romania; 4 Clinic of Internal Medicine, Dr. I. Cantacuzino Clinical Hospital, Bucharest, Romania; 5 Carol Davila University of Medicine and Pharmacy, Bucharest, Romania; 6 Clinic of Pathology, Colțea Clinical Hospital, Bucharest, Romania; 7 PathoTeam Diagnostic Laboratory, Bucharest, Romania

**Keywords:** Hodgkin lymphoma, microenvironment, prognostic factors, immunohistochemistry (IHC), Epstein-Barr virus (EBV)

## Abstract

Hodgkin lymphoma (HL) has become one of the most curable hematological neoplasia. Clinical and biological factors remain the main pillars guiding therapeutic strategies in HL. Recent studies have improved our understanding of the phenotype, the characteristics of histogenesis, and other possible mechanisms of lymphomagenesis, including the role of Epstein-Barr virus (EBV) infection. Tumor cells manipulate the microenvironment, allowing them to develop their malignant phenotype and evade the attack of the host's immune response so that the interaction between tumor cells and the reactive microenvironment determines not only the histological features but also the clinical-pathological characteristics and prognosis of these patients - essential for the development of future therapies targeting various other cellular components of the tumor microenvironment. This article aimed to evaluate the characteristics of the tumor microenvironment and malignant cells using histopathology and immunohistochemistry (IHC) techniques to highlight the association of EBV and to study the expression of characteristic antigens in malignant and non-malignant cells within the tumor mass (overexpression of BCL2 (B-cell lymphoma 2) in malignant cells, presence of PD1 (Programmed cell death Protein 1) on T lymphocytes, CD68+ macrophages in the tumor microenvironment, and presence of EGFR (epidermal growth factor receptor). The analysis of the data collected in this paper highlights several key parameters with prognostic value and statistical significance: the EBV infection at diagnosis, its association with low-intensity BCL2(+), the presence of CD68 with rosette formation, and the identification of specific vascularization patterns. The development of prognostic systems that take into account the integration of biological prognostic markers seems essential for a better risk stratification.

## INTRODUCTION

Classical Hodgkin lymphoma (cHL) represents an important pathology of young adults, constituting 11% of all lymphoma diagnoses. Current therapeutic strategies, which typically combine cytostatic chemotherapy with radiotherapy, have significantly improved the outcome of cHL, with high cure rates, even in advanced stages [1, 2]. Nevertheless, a subset of patients, 15-20% with early-stage disease - stages I and II, as well as 15-30% of those in more advanced stages - III and IV - demonstrate resistance to first-line therapy or relapse [3].

The current method for devising therapeutic strategies primarily relies on prognostic scoring systems, which solely incorporate clinical indicators, standard laboratory findings, and diagnostic imaging data. The factors accounted for in these scores include the stage of the disease, the patient's age, disease-specific biomarkers, hemoglobin levels, leukocyte count, eosinophils, lymphocytes, serum albumin concentration, and erythrocyte sedimentation rate [4, 5]. The present paradigm for patient stratification, which overlooks the tumor process' unique characteristics in cHL, lacks sufficient predictive value for disease progression in a substantial subset of cases. This limitation is most evident in the scarcity of patient-specific therapeutic approaches.

Consequently, therapeutic modalities for cHL could inadvertently result in under- or over-treatment. The application of cytotoxic chemotherapy, especially when synergized with radiotherapy, can contribute to both short and long-term effects, potentially impairing survival rates due to secondary neoplasia, cardiac dysfunction, immunosuppression, and endocrinological disturbances [6]. Consequently, current hematological investigations focus on delineating prognostic markers linked to tumorigenic attributes to facilitate risk-specific therapeutic adaptations.

Classical Hodgkin Lymphoma presents a unique oncological entity, characterized by the fact that the malignant Reed-Sternberg cells (CRS) constitute a mere 2% of the tumor mass. The remainder comprises a heterogeneous population of non-malignant cells, forming a robust inflammatory infiltrate. This complex microenvironment, orchestrated by the CRS cells, promotes their survival and protection from constant immune system surveillance [2]. Recent research data, also addressed by us in this article, have identified a number of immunophenotypic and genomic characteristics of CRS with prognostic significance: expression of BCL2, insertion of Epstein-Barr virus (EBV) genome, and increased expression of some genes related to increased treatment resistance. The incorporation of cells within the microenvironment is managed by CRS via the release of cytokines and chemokines. The serum concentrations of some of these entities may hold prognostic implications in the progression of the disease [5]. Non-malignant cells in the tumor microenvironment also contribute to prognosis. Studies have shown that the proportion of non-malignant cells, their phenotypical characteristics, and the gene expression pattern impact the response to treatment [7].

Several studies demonstrated the potential role of various biomarkers in refining current prognostic indexes [5-7]. Incorporating these biomarkers into novel prognostic models based on RSC phenotypic and genetic characteristics and microenvironment interactions could improve patient risk stratification and prediction of therapeutic outcomes.

This study aimed to examine the properties of the tumor microenvironment and malignant cells through methodologies that are readily accessible or can be successfully and relatively easily established in hematology-oriented laboratories nationwide. Histopathological and immunohistochemical (IHC) techniques were employed for phenotypic characterization of the tumor microenvironment and malignant cells. This includes detecting EBV association, as well as studying the expression of antigens characteristic of malignant and non-malignant cells within the tumor-specific niche.

## MATERIAL AND METHODS

The study group included 93 patients with confirmed diagnosis of classical Hodgkin lymphoma from our clinic between 2005-2018, which were analyzed taking into account a number of clinical and paraclinical parameters with a proven prognostic role, as well as potential prognostic factors correlated with the evolution of the disease, noted in dynamics.

All patients signed a consent form involving the use of data from the Observation Sheet for research/ publication purposes, and the study received approval from the Ethics Commission of the institution.

The study started in 2016 as a retrospective/prospective analytical observational study and included patients with available data on clinical, biological, and therapeutic response parameters with already proven prognostic value:

- factors related to the patient (age, sex, clinical performance status)
- parameters assessing tumor mass (presence of B symptoms, Ann Arbor stage, number of affected ganglion areas, presence of bulky tumor masses, type and number of extra-nodal determinations, presence of medullary determination, presence of hepatomegaly/ splenomegaly, serum level of lactate dehydrogenase (LDH), serum level of beta-2-microglobulin)
- prognostic factors related to the biology of the disease - histological subtype, biological parameters (complete blood count, iron level, ESR level, serum albumin level)
- prognostic factors related to treatment (type of chemotherapy used in induction, use of radiotherapy, monoclonal antibodies, transplantation (autologous) of hematopoietic stem cells, need for intermediate positron emission tomography and computed tomography scan (PET-CT)

Patients diagnosed with cHL with all four histological subtypes (according to WHO classification) - Nodular Sclerosis (NS), Mixed Cellularity (MC), Lymphocyte-rich (LR), Lymphocyte-depleted (LD) - were consecutively included in this analysis. The follow-up period was until December 2019 or until death occurred.

Patients lost from observation during the study and patients diagnosed with a nodular variant of Hodgkin lymphoma were excluded from the analysis.

All patients received first-line polychemotherapy regimens - ABVD, BEACOPP - according to international treatment guidelines and protocols.

In order to achieve clinical-histological correlations, the group was subdivided, depending on the response to treatment at the end of the study, into 2 subgroups:

- Subgroup 1 (Lot I) consisted of 58 patients with favorable disease evolution – in complete remission after the first line of treatment.
- Subgroup 2 (Lot II) consisted of 35 patients with unfavorable evolution of disease or refractory cases – in which we introduced patients in partial remission, patients with progressive disease, as well as patients with stable disease after the first line of treatment.

The analysis of new prognostic factors that could assess the course of the disease was performed in 34 of those 93 initially collected cases by conducting a prospective study:

- presence of Epstein-Barr virus;
- overexpression of B-cell lymphoma 2 (BCL2) in malignant cells;
- presence of Programmed cell death Protein 1 (PD1) on T lymphocytes;
- CD68+ macrophages in the tumor microenvironment;
- presence of epidermal growth factor receptor (EGFR) – regarding the stimulation of angiogenesis.

These parameters were assessed by immunohistochemical staining performed on sections of tissue material embedded in paraffin. We used the following antibody panels:

- A panel of 5 antibodies to establish the immunophenotypic diagnosis of cHL: CD30, CD20, PAX5, CD3 (the source: Ventana, Roche Diagnostics, Arizona, USA), and CD15 (the source: Leica Biosystems, Buffalo Grove, USA);
- An additional panel of 6 antibodies to investigate their expression in the tumor cells and the peritumoral microenvironment: EBV/LMP1 (the source: Leica Biosystems, Buffalo Grove, USA), BCL2 (the source: Ventana, Roche Diagnostics, Arizona, USA), PD1 (the source: BioSB, Santa Barbara, USA), EGFR (the source: Sigma Aldrich, Merck KgaA, Darmstadt, Germany) and CD68 (the source: BioSB, Santa Barbara, USA).

Immunohistochemical staining was performed by automatic and manual staining techniques:

a) Automatic immunohistochemical staining technique (according to the manufacturer's indications) was performed using two types of immunostaining:

- Bench Mark Ultra-Stainer Module immune-Stainer (Ventana, Roche Diagnostics) for anti-CD20, CD3, PAX5, BCL2 antibodies (antigen-antibody reaction was highlighted using Ultra View Universal DAB detection Kit) and anti-CD30 antibodies (antigen-antibody reaction was highlighted using Opti View DAB IHC Detection Kit and Opti View Amplification Kit);
- Bond-III immune-Stainer (Leica Biosystems) for anti-CD15 antibodies (highlighting the reaction was done with the Bond Polymer Refine Detection Kit).

b) Manual staining technique used for anti-EBV/LMP1, PD1, EGFR and CD68 antibodies

*Manual immunohistochemical staining technique:*

- the histological sections included were dewaxed, rehydrated, and washed in phosphate buffer saline (PBS) solution, pH = 7.4;
- unmasking antigenic sites by heat treatment (boiling in microwave oven in EDTA pH-9 buffer solutions);
- incubation with specific antibody at room temperature, 1 hour;
- incubation with Ultra Vision Quanto Detection System HRP DAB polymer for 30 minutes at room temperature;
- development in 3-3’ diaminobenzidine (DAB) for 5-10 minutes;
- counterstaining with Hematoxylin Mayer for 2-3 minutes;
- dehydration, clarification, and mounting of blades.

For both automatic and manual techniques, development was done with 3-3’ diaminobenzidine (DAB), which gives a brown precipitate.

*Reaction assessment for additional antibody panel:*

- the reaction for EBV/LMP1 in tumor cells RS was noted by "+", respectively "-", without the percentage quantification of the number of positive tumor cells (qualitative reaction);
- the reaction for BCL2 in RS tumor cells was noted by "+", respectively "-"; for "+" cases, a qualitative assessment of the intensity of the reaction was made (on a scale from + to +++, relative to the BCL2 reaction in small reactive lymphocytes) and a quantitative assessment of the percentage of positive BCL2 tumor cells;
- the reaction for PD1 was noted in the peritumoral microenvironment, appreciating, quantitatively, the percentage of PD1+ small lymphocytes and their disposition in relation to the RS tumor cells (possible peritumor rosette);
- the reaction for CD68 was noted in the peritumoral microenvironment, appreciating, quantitatively, the percentage of CD68+ cells and their disposition relative to the RS tumor cells (possible peritumor rosette);
- reaction for EGFR: five fields with the highest vascular density were selected for each section (selected with 10x lens, 20x eyepiece with Leica DMC 2900 microscope). These were captured using a digital camera with Leica Application Suite (LAS) software. The vessels marked by the EGFR reaction for each field were counted, and an average was made.

Data analysis was conducted using the Statistical Package for the Social Sciences (SPSS) software (the square chi-square test and p-value statistical materiality threshold). Most of the variables used in the analysis were measurable biological parameters, but in some cases, binary categorical values were introduced due to the need to capture qualitative phenomena or characteristics. For variables with more than two values, their transformation into primary variables was also considered. In the present paper, the chi-square test was used to compare patients according to disease evolution and the influence of prognostic factors on favorable or unfavorable disease evolution.

## RESULTS

The main prognostic factors and their impact on the evolution of Hodgkin lymphoma were analyzed (Table 1).

**Table 1:** General characteristics of study subgroups

Characteristics	Results	
	Lot I	Lot II
**Age (no. patients)** average (years) limits	36.47 18 - 69	40.61 18 - 78
**Sex (no. patients)** male/ female M/F report	31/ 27 1.15/ 1	20/ 15 1.33/ 1
**Environment (no. patients)** urban/ rural	42/ 15	19/ 17
**ECOG status (no. patients)** 0/ 1/ 2/ 3/ 4	45/ 10/ 2/ 0/ 1	19/ 11/ 5/ 0/ 0
**Stage (no. patients)** I/ II/ III/ IV	2/ 18/ 19/ 18	1/ 5/ 6/ 24
**B symptoms (no. patients)** Present/ Absent	26/ 32	26/ 9
**Histological subtype** **(no. patients)** NS/ MC/ LR/ LD	31/ 22/ 1/ 4	14/ 19/ 0/ 2
**Onset extranodal determination (% patients)** Present/ Absent	41.38/ 58.62	74.29/ 25.71
**Iron level (% patients)** Normal/ Low	51.72/ 48.28	31.43/ 68.57
**Hemoglobin level** **(% patients)** Normal/ Low	54.39/ 45.61	47.22/ 52.78
**Leukocyte level** **(% patients)** **Normal/ High**	63.79/ 36.21	54.29/ 45.71
**Lymphocyte level** **(% patients)** Normal/ Low	51.72/ 48.28	42.86/ 57.14
**ESR level (% patients)** Normal/ High	32.76/ 67.24	11.11/ 88.89
**Serum level of LDH** **(% patients)** Normal/ High	56.90/ 43.10	62.86/ 37.14
**Serum albumin level** **(% patients)** Normal/ Low	81.03/ 18.97	71.43/ 28.57
**Serum level of beta-2-microglobulin (% patients)** Normal/ High	63.79/ 36.21	45.71/ 54.29

Lot I=Subgroup 1; Lot II=Subgroup 2; M=male; F= female; NS=nodular sclerosis; MC=mixed cellularity; LR-lymphocyte rich; LD=lymphocyte depletion; ESR=erythrocyte sedimentation rate; LDH=Lactate dehydrogenase

The majority of patients received as first-line therapy the ABVD protocol (68.82%) after diagnosis, and only one-third - BEACOPP regimen (31.18%). Among the entire patient cohort, approximately two-thirds of patients (61.29%) achieved complete remission after the first line of therapy, while 16.13% achieved partial remission. 19.35% of cases progressed despite treatment, and 3.23% remained stationary.

The analysis of new prognostic factors that could assess the evolution of the disease was performed through a prospective study analyzing 34 cases of a total of 93 patients, as presented in Table 2.

**Table 2:** Characteristics of patients according to new potential prognostic factors

Patient	EBV 1=present 0=absent	BCL2 0=abs 1= + 2= ++ 3= +++	BCL2 (%)	PD1 (remote/ rosette)	PD1 (%)	Mfg CD68+ (remote/ rosette/ proximity)	Mfg CD68+ (%)	EGFR (no.vessels) *could not exactly be counted
1	0	3	90	remote	40	rosette	40	52
2	1	1	30	remote	10	rosette	40	62
3	0	1	30	remote	10	remote	10	28
4	1	0	-	rosette	30	remote	30	44
5	1	3	50	remote	30	remote	10	74
6	1	2	80	rosette	60	remote	10	48
7	0	1	30	remote	10	remote	30	*
8	1	1	30	rosette	50	remote	25	48
9	1	0	-	remote	30	proximity	25	*
10	1	0	-	remote	10	proximity	35	44
11	1	1	20	remote	20	remote	10	*
12	1	0	-	remote	30	remote	20	*
13	1	0	-	remote	10	remote	20	*
14	1	0	-	remote	10	proximity	40	25
15	1	0	-	remote	30	remote	10	*
16	1	0	-	remote	10	proximity	30	67
17	1	0	-	rosette	60	remote	10	78
18	0	2	50	remote	20	remote	10	*
19	1	0	-	remote	10	remote	30	*
20	1	3	90	remote	10	remote	10	*
21	1	1	30	remote	10	remote	10	*
22	1	1	30	remote	10	remote	10	*
23	0	0	-	remote	10	remote	20	*
24	1	1	30	remote	10	proximity	30	24
25	0	2	75	remote	10	proximity	30	*
26	1	1	60	remote	10	proximity	40	21
27	0	3	90	rosette	40	rosette	40	*
28	0	0	-	rosette	30	remote	10	42
29	0	1	30	remote	10	remote	10	*
30	1	0	-	remote	10	remote	25	*
31	1	3	90	rosette	60	remote	25	54
32	0	2	50	remote	10	rosette	40	25
33	1	3	90	remote	10	remote	10	*
34	0	3	90	remote	10	remote	10	*

EBV=Epstein-Barr virus; BCL2=B-cell lymphoma 2; PD 1=Programmed cell death Protein 1; Mfg=macrophages; EGFR=epidermal growth factor receptor

### Epstein-Barr virus impact

One-third of the patients in the overall study group underwent testing for Epstein-Barr virus (EBV) infection. Among those tested, there were approximately twice as many individuals with EBV-positive results compared to those with negative results (24.73% *vs*. 11.83%). The majority of cHL patients tested for EBV were men, both among positive cases of EBV (14 cases) and among negative ones (9 cases).

Analyzing the obtained data, we observed a significantly increased distribution of cHL-NS EBV (+) cases compared to negative ones (13 compared with 5 cases). A similar pattern was evident for cHL-MC cases (10 cases compared to 4), and in the case of the other 2 histological subtypes (LR, LD), 1 case with EBV (-) was recorded for each. At the same time, comparing the cases of EBV (+) according to the histological subtype, a relatively high proportion of patients with NS was detected compared to those with MC.

**Figure 1:**
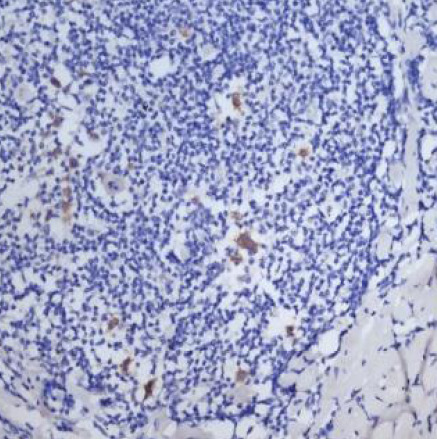
Case no.8 – cHL-NS, EBV/LMP1 positive in tumoral cells EBV/LMP1, ob 20%)

**Figure 2:**
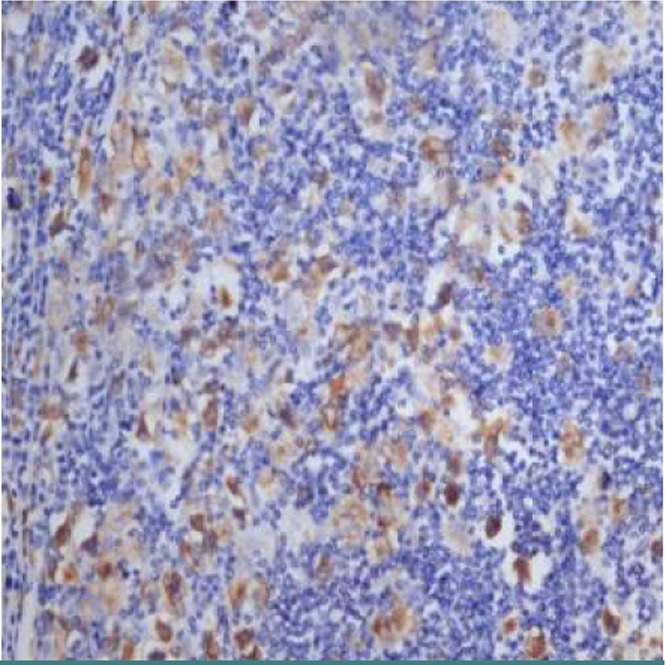
Case no.15 – cHL-MC, EBV/LMP1 positive in tumoral cells (IHC reaction for EBV/LMP1, ob 20x)

In 23 of the studied cases (approximately 68% of the cases further analyzed), we could evaluate tumor cells for EBV/LMP1 (latent membrane protein 1) expression; the reaction was diffuse cytoplasmic, more pronounced paranuclear granular in the Golgi apparatus (Figures 1-2).

**Figure 3:**
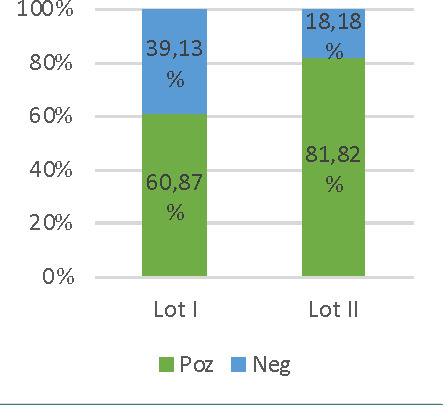
Distribution by samples of patients who have been tested for EBV infection

Although in both subgroups, the share of patients with EBV (+) was consistent, it can be observed (Fig. 3) that the percentage of positive patients in subgroup 2 (81.82%) was higher than the one of positive cases in subgroup 1 (60.87%). Furthermore, looking at the second subgroup of patients with unfavorable disease evolution, a significantly higher rate of positive cases (81.82%) can be observed compared to negative cases (18.18%). This fact highlights the negative impact of EBV infection in the evolution of patients with Hodgkin lymphoma and the statistically significant association (p=0.021895) (Fig 3).

### Overexpression of BCL2 in malignant cells

BCL2 expression was assessed in 21 cases (61.76%). The percentage of BCL2(+) tumor cells was variable, between 20% and maximum 90%.

**Figure 4:**
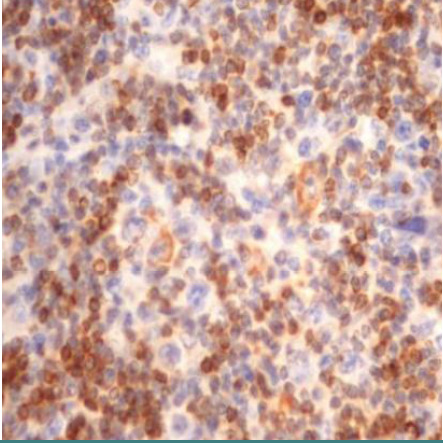
Case no.29, cHL-NS – BCL2 positive (+) in RS cells (IHC staining for BCL2 ob 20x)

**Figure 5:**
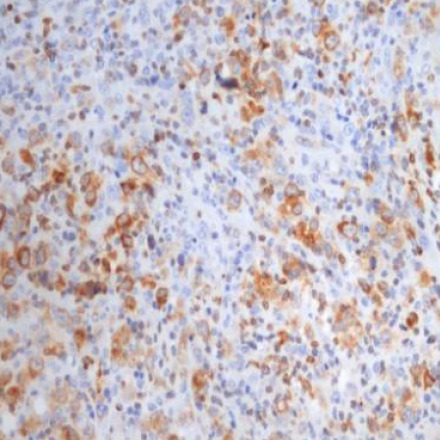
Case no.33, cHL-NS – BCL2 positive (+++) in RS cells (IHC staining for ob 20x)

**Figure 6:**
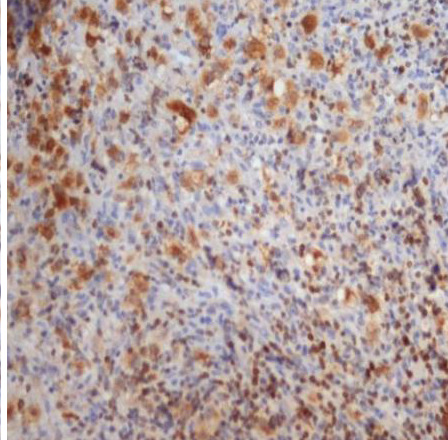
Case no.18, cHL-NS – BCL2 positive (++) in RS cells (IHC staining for BCL2 ob 20x)

Compared to the BCL2 reaction in small reactive lymphocytes, the expression of BCL2 in Reed-Sternberg cells was of low intensity (noted “+”) in 47.62% of BCL2(+) (Fig. 4), very intensely positive cases (noted “+++”) in approximately 33% of cases (Fig. 5) and the same intensity with the BCL2 reaction in small lymphocytes (noted “++”) in the remaining ~19% of cases (Fig. 6) (the comparison was made for the highest reaction intensity). Overall, an increased response intensity (+++) for BCL2 was correlated with a high percentage (90%) of positive RS cells.

### PD1 expression on T lymphocytes

**Figure 7:**
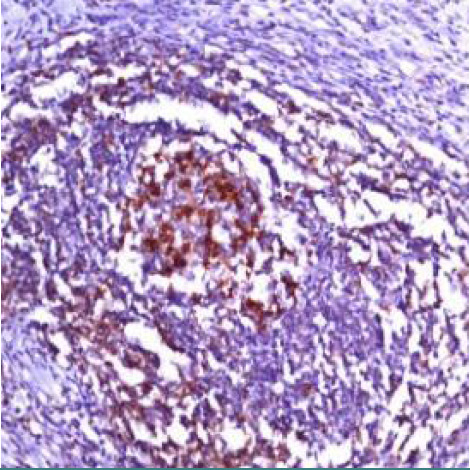
Case no.18, cHL-NS – lower reaction intensity for peritumor PD1 compared to germination centers (IHC staining for PD1, ob 10x)

**Figure 8:**
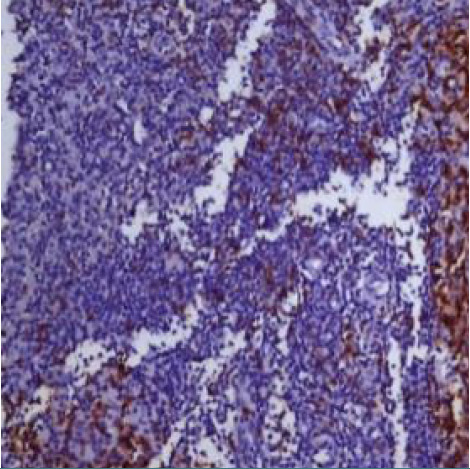
Case no.3, cHL-MC – very rare, <10%, PD1 positive lymphocytes (IHC staining for PD1, ob 10x)

**Figure 9:**
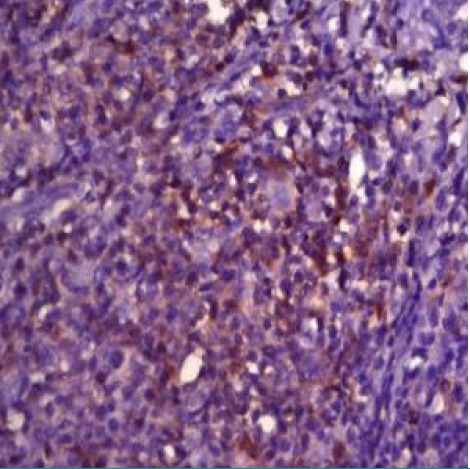
Case no.6, cHL-MC, frequent positive PD1 cells, which rosette around RS cells (IHC staining for PD1, ob 20x)

**Figure 10:**
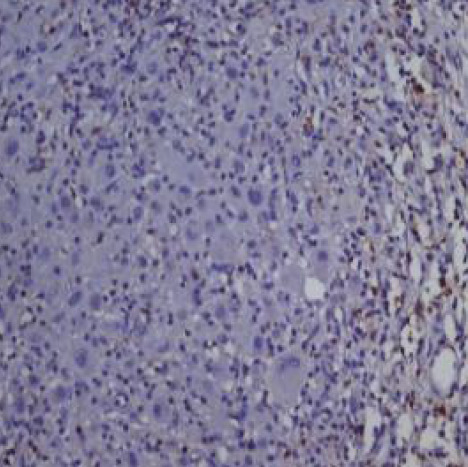
Case nr.1 – cHL-LD – very rare positive PD1 cells in areas with frequent RS cells (IHC staining for PD1, ob 20x)

The reaction for PD1 in the peritumoral microenvironment was of lower intensity compared to the reaction in small follicular T helper lymphocytes in the remaining reactive germ centers (Fig. 7). In most cases studied (20 cases, 60%), the percentage of low PD1 follicular T helper lymphocytes in the peritumoral microenvironment were reduced (<10%) (Fig. 8). In rare cases (5 cases, 15%) the percentage of small PD1 positive lymphocytes was between 30 and 50% of cells, with rosette form arrangement around tumor cells (Fig. 9). It is important to highlight the very low number of small T helper follicular lymphocytes in high-density areas of tumor cells (Fig. 10).

Most patients (~80%) had a remote PD1 presence in the tumor microenvironment. In terms of sample distribution, there was an insignificant increase in patients in the second subgroup compared to those in the first subgroup, according to the presence of remote PD1 and, surprisingly, a low share of patients with unfavorable disease evolution compared to those with favorable evolution in case of PD1 with rosette, without statistical significance (p=0.810339).

### CD68+ macrophages in the tumor microenvironment

**Figure 11:**
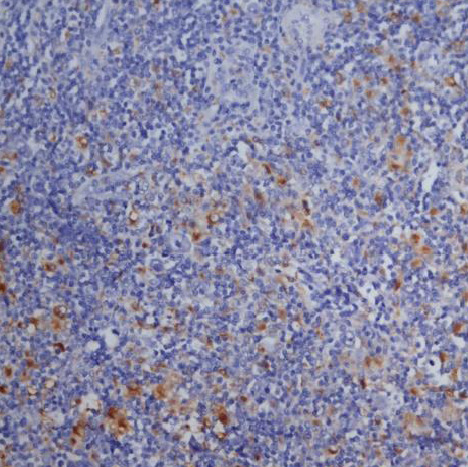
Case no.15, cHL-MC, rare positive CD68 cells, at distance from RS cells (IHC staining for CD68, ob 20x)

**Figure 12:**
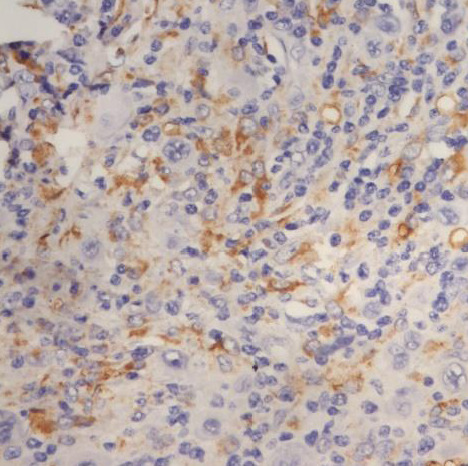
Case no.1, cHL-LD, frequent CD68 positive cells, incomplete rosary around RS cells (IHC staining for CD68, ob 40x)

The percentage of histiocytes and macrophages in the peritumoral microenvironment was variable between 10% and 40% of cellularity. In cases with a lower percentage of positive CD68 cells (Fig. 11), these cells were arranged at a distance from the RS cells; if the percentage of positive CD68 cells was high (40-50%), they were also arranged in the proximity of the RS cells, sometimes (3 cases – 12%) with incomplete rosette around them (Fig. 12). The analysis of the cases showed a relatively equal distribution in the proportion of patients with CD68+ cells in the microenvironment located at distance from RS cells according to the evolution of disease (69.57% and 63.64%) with p>0.05 and a higher proportion of patients with CD68+ cells with rosette in the second subgroup (patients with unfavorable prognostic) compared to the first subgroup (p<0.05).

### Stimulation of angiogenesis

**Figure 13:**
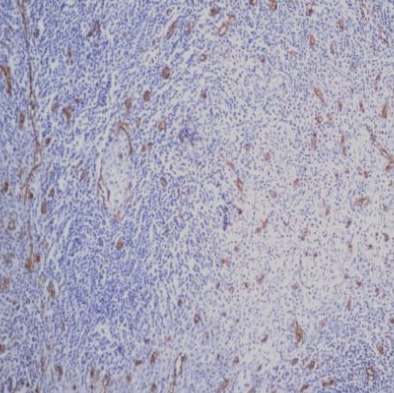
Case no.17, cHL-NS, greater density of vessels in peritumor sclerosis (IHC staining for EGFR, ob 10x)

**Figure 14:**
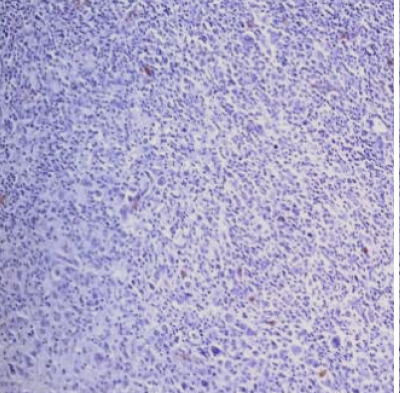
Case no.1, cHL-LD, very rare vessels in areas with high density of tumor cells (IHC staining for EGFR, 10x)

**Figure 15:**
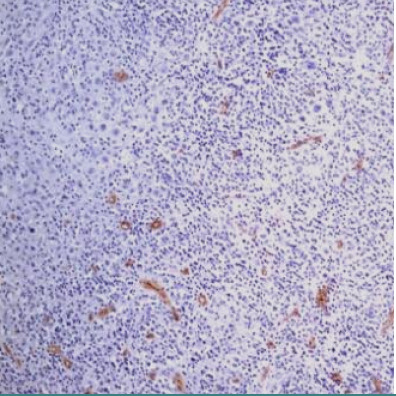
idem – frequent vessels in areas with rare tumor cells (IHC staining for EGFR, ob 10x)

The number of vessels was variable, between 21 vessels and 78 vessels. In instances of cHL-SN, the vascular density was higher in peritumoral sclerosis compared to areas of tumor infiltration (Fig. 13). It is noteworthy that within the same case, there were lower number of vessels in areas with high tumor cell density (Fig. 14) compared to areas with rare tumor cells (Fig. 15). This observation supports the argument that neo- angiogenesis is an important factor of tumor growth in cHL, correlated with the higher percentage of refractory cases in the second group compared to the first group (p<0.05), both regarding number of vessels less than 50 and also greater than 50 vessels.

## DISCUSSION

Hodgkin lymphoma has become one of the most possible curable hematological neoplasia, both in adult patients and especially in those of pediatric age with a current high general rate of cure.

The microenvironment is a fundamental component of the tumor mass and an essential pathogenetic factor in Hodgkin lymphoma, providing tumor cells with growth factors and inhibiting antitumor immune responses.

The continuous study of the Hodgkin-Reed Sternberg cells characteristic of this type of lymphoma, as well as especially the analysis of the surrounding tumor microenvironment, especially in the case of patients with advanced diseases, were the basis of various research activities that helped to understand Hodgkin lymphoma from a pathophysiological, immunological and molecular point of view, but also on the relevance of EBV infection and the release of cytokines in the case of at least a proportion of patients.

Tumor cells manipulate the microenvironment, allowing them to develop their malignant phenotype and evade the attack of the host's immune response, so the interaction between the tumor cells and the reactive microenvironment determines not only the histological features but also the clinical-pathological characteristics and prognosis of these patients.

BCL2 is a protein with antiapoptotic properties that plays a significant role in tumorigenesis. It is noteworthy that, unlike typical oncogenes that exert their oncogenic influence by promoting cell proliferation, BCL2 functions by inhibiting programmed cell death. As a result, BCL2 overexpression has been considered a promising prognostic marker in various neoplastic conditions [8].

The existing literature presents conflicting findings regarding the prognostic significance of BCL2 in various human malignancies. Some researchers have reported a correlation between BCL2 expression and decreased disease-free survival (DFS) and overall survival (OS) [8]. However, other investigations have indicated a weak statistical correlation between BCL2 protein expression and OS, event-free survival (EFS), or treatment refractoriness [9-11]. It is worth noting that the absence of BCL2 expression may also have implications, as it suggests a switch in the survival pathway of the expressing cell, potentially associated with a more aggressive disease phenotype [9].

In our study, we observed a significant population exhibiting reduced intensity (+) expression of BCL2 (p<0.05). However, we did not find a statistically significant association between the expression of BCL2 protein and the unfavorable progression of the disease (p= 0.076578), aligning with the findings reported in more recent publications.

Programmed death-1 (PD-1) is a protein expressed on various immune cells, including B cells, NK cells, dendritic cells, activated monocytes, and particularly on activated T cells [12]. It serves as an immune checkpoint molecule that regulates the activation and proliferation of T cells. Upon binding with one of its known ligands, PD-L1, the PD-1 receptor initiates a potent inhibitory signal that leads to reduced cytokine production and decreased proliferation. As a result, the PD-1/PD-L1 signaling axis plays a critical role in tumor biology, the tumor microenvironment, and the evasion of immune surveillance mechanisms by cancer cells [12, 13].

In our study, we conducted a comparison of PD-1 (+) staining intensity in the tumor microenvironment in relation to the small follicular T helper lymphocytes present in the adjacent reactive germ centers. Our findings indicate that the intensity of PD-1 staining in the tumor microenvironment is generally lower (e.g., case nr.18 - NS) compared to the staining observed in small follicular T helper lymphocytes.

Furthermore, there is notable variability in the percentage of PD-1 lymphocytes in the peritumoral microenvironment. Most cases (60%, 20 cases) exhibited a percentage of PD-1 lymphocytes of less than 10%. In contrast, a smaller subset of cases (15%, 5 cases) demonstrated a higher percentage ranging between 30% and 50%. Interestingly, the cases with a high concentration of PD-1 positive lymphocytes also exhibited a distinct rosette-like arrangement surrounding the tumor cells. Most patients (80%) exhibited a remote PD-1 tumor microenvironment (TME). However, our analysis revealed that the increase observed in the second group compared to the first group was not statistically significant. Surprisingly, a lower proportion of patients with unfavorable disease progression was found in cases where PD1 was present with a rosette formation around the tumor cells, but this association did not reach statistical significance (p= 0.810339).

In one of the studies on cHL, a high abundance of PD-1(+) lymphocytes served as a negative prognostic factor for OS independent of the disease stage [14]. Furthermore, evidence suggests that cases with a high infiltration of PD-1(+) tumor-infiltrating macrophages and co-expression of PD-1 and PD-L1 are associated with an inferior clinical outcome [12, 15]. The signaling function of PD-1 plays a crucial role in the pathogenesis of Hodgkin lymphoma by contributing to the phenomenon known as "T-cell exhaustion." This condition involves the loss of T lymphocytes' ability to effectively eliminate target cells [8]. In certain multivariate analyses, a high proportion of PD-1(+) lymphocytes has been associated with reduced EFS [15, 16].

The tumor microenvironment (TME) is a specialized cellular niche formed through the dynamic cytokine interactions between the tumor cells and surrounding components of the peri-tumoral environment [17]. This intricate interplay between the tumor and its microenvironment critically influences the morphological characteristics and clinical outcome of the disease. Tumor-associated macrophages (TAMs) are a cellular subset known to play a crucial role in shaping the tumor microenvironment. TAMs can exhibit diverse phenotypes, including the M1 phenotype characterized by the expression of CD68, and the M2 phenotype characterized by the expression of CD163 [18]. The literature extensively reports that overexpression of CD68+ cells in the tumor microenvironment is associated with decreased progression-free survival (PFS), overall survival (OS), and increased risk of relapse [19-22]. Different hypotheses suggest that macrophages promote genetic instability within the tumor microenvironment, thereby impacting patient survival [23-25].

In our microenvironment samples, we observed a variable population of macrophages, ranging from 10% to 40% of the total cellularity. Interestingly, we noticed a distinct pattern of arrangement for these cells. As the population of macrophages decreased, they were located at a greater distance from the RS cells. Conversely, with a higher percentage of macrophages, they were found in closer proximity to the RS cells. In a subset of cases (12%), these macrophages formed incomplete rosettes around the RS cells. The data analysis reveals a comparable distribution of patients with CD68+ cells located at a distance from RS cells between different disease progression groups (69.57% and 63.64%). However, a higher proportion of patients in the second group exhibited CD68+ cells forming rosettes compared to the first group (p<0.05), our findings confirming the evidence cited in the literature.

Angiogenesis, the process of forming new blood vessels from pre-existing vasculature, plays a critical role in the progression of hematological malignancies and solid tumors. The development of a neoplasm typically involves two distinct phases. Initially, during the avascular phase, the developing tumor relies on the readily available nutrients provided by the existing blood vessels. Subsequently, in the vascular phase, tumor cells can induce the formation of new blood vessels (neo-vasculature) to support their continued progression [26]. The transition between the two states is commonly referred to as the “angiogenic switch” [27], caused by the shift in equilibrium between the pro-angiogenic molecules and anti-angiogenic molecules.

One of the more extensive studies on HL angiogenesis is the investigation of vessels by the morphometric approach [28]. The study findings demonstrated a notable increase in blood vessel caliber with advancing Ann Arbor stages. This observation provides evidence for a correlation between a larger microvessel lumen and an increased likelihood of tumor cell access to the circulation [28]. The literature is abundant in studies investigating the roles of different angiogenic cytokines in different HL subtypes. The significant overexpression of VEGF-D in HRS cells, particularly in cases with high tumor microvessels, strongly indicates its critical role in angiogenesis and the tumor microenvironment [29]. Also, VEGFR-1 and VEGFR-2, two tyrosine kinase receptors, bind to VEGF-A, activating survival pathways in endothelial cells, such as the Raf-MEK-MAP pathway. This activation promotes the survival and proliferation of endothelial cells [30, 31]. The study examining the expression patterns of these molecules revealed a correlation between their expression levels and neoplastic vessel branching [32-34].

We found that vascular density in the tumor microenvironment varied between 21 and 78 vessels. In cHL-SN cases, peritumoral sclerosis exhibited higher vascular density compared to tumor-infiltrated areas (Fig. 13). Notably, areas with high tumor cell density had fewer vessels compared to areas with rare tumor cells (Figures 14 and 15). This suggests that neo-angiogenesis plays a crucial role in cHL tumorigenesis. Furthermore, a significant correlation was observed between neo-angiogenesis and a higher percentage of refractory cases in the second group (p<0.05), regardless of vessel count (less than 50: 45.45% *vs*. 21.74%; greater than 50: 27.27% *vs*. 13.04%), confirming the strong correlation between angiogenic patterns and different clinical outcomes.

There is a relationship between Hodgkin lymphoma and EBV infection, as shown by data from the literature [35, 36]. The mechanisms underlying this association are unknown, but studies have shown that patients with a history of infectious mononucleosis caused by Epstein-Barr virus may be at a higher risk of developing Hodgkin lymphoma. Consequently, EBV stands out as the primary implicated virus in the development of Hodgkin lymphoma [37]. Nevertheless, the presence of the EBV genome within the tumor has been identified in only about 20-40% of Hodgkin lymphoma cases previously diagnosed with infectious mononucleosis [38]. Several studies suggest that EBV may be a transformative agent in Hodgkin lymphoma. Patients with an infectious history of EBV are 2-3 times more likely to develop Hodgkin lymphoma [39].

The majority of patients with cHL tested for EBV were men, both among positive cases of EBV and in negative cases, as specified in the literature. There was a significantly increased distribution of cHL-NS EBV-positive cases compared to negative ones (72.22% *vs*. 22.78%), a similar situation for cHL-MC cases (71.43% compared to 28.57%), and for the other 2 histological subtypes (Lymphocyte-rich, Lymphocyte-depleted) just 1 case with EBV (-). At the same time, comparing EBV (+) cases according to the histological subtype, a relatively high proportion of patients with NS were detected compared to those with MC. The latest obtained data correlates only partially with those in the literature, the latter claiming that most cases of cHL-MC are EBV-positive (75%), but the majority of cases of cHL-NS are EBV-negative, with reported positivity rates varying between 10-40%.

The limitation of the study lies in the retrospective component of the analysis and the small number of patients in the prospective part. The prospective component allowed the analysis of cases from a perspective that focused on parameters reflecting the biology of the tumor process (overexpression of BCL2 in malignant cells, presence of PD1 on T lymphocytes, CD68+ macrophages in the tumor microenvironment, and the presence of EGFR with regards to its role in angiogenesis stimulation.

## CONCLUSION

The data analysis from this study emphasized parameters with prognostic value and statistical significance. These include a range of factors, such as low iron level, biologic inflammatory syndrome (ESR), increased serum beta-2-microglobulin level, onset extra nodal determination, ECOG performance status, EBV infection at diagnosis, association with low-intensity BCL2(+), the presence of CD68 with rosette, and distinct vascularization patterns. On the other hand, certain parameters had prognostic value but lacked statistical significance, including the onset anemia and/ or leukocytosis, early lymphopenia, LDH level, hypoalbuminemia, diverse grades of BCL2 expression (++ and +++), the presence of PD1, and the association of CD68 in relation to distance and proximity. To validate the results obtained so far, it would be necessary to conduct prospective studies, with the possibility of expanding the investigation panel and enrolling larger groups of patients. The widespread use of new biological and molecular prognostic factors in diagnostics, highly effective new molecules, and recent knowledge of chemotherapeutic effects on the microenvironment would allow for a different and personalized therapeutic approach to Hodgkin lymphoma in the short and long term.
